# The effects of aspirin plus cisplatin on SGC7901/CDDP cells *in vitro*

**DOI:** 10.3892/br.2014.241

**Published:** 2014-02-26

**Authors:** HANZHANG DONG, GAOGAO LIU, BIAO JIANG, JIUBING GUO, GUOQUAN TAO, WEI YIU, JINGSONG ZHOU, GUOXIN LI

**Affiliations:** 1 Department of General Surgery, The Affiliated Nanfang Hospital of Southern Medical University, Guangzhou, Guangdong 510515, P.R. China; 2 Department of General Surgery, The Affiliated Beijiao Hospital of Southern Medical University, Shunde, Guangdong 528311, P.R. China

**Keywords:** cisplatin resistance, survivin, gastric cancer, chemotherapy

## Abstract

The purpose of this study was to determine the effect of aspirin plus cisplatin (CDDP) in the chemotherapy of gastric cancer. We cultured SGC7901/CDDP cells by long-term exposure of SGC7901 cells to small doses of CDDP *in vitro*. The cells were treated with aspirin, CDDP or aspirin plus CDDP for 24 h and cell growth was assessed by the MTT assay, the apoptotic rate by flow cytometry, the survivin mRNA expression by RT-PCR and the survivin protein expression by western blotting. The results revealed that the cell growth in the aspirin plus CDDP group was significantly inhibited. The apoptotic rate in the aspirin plus CDDP was significantly higher compared to that in the other groups. The survivin mRNA and protein expression were also significantly reduced in the aspirin plus CDDP group. Our data suggest that the combination of aspirin and CDDP exhibited a higher degree of toxicity against SGC7901/CDDP cells compared to that of aspirin or CDDP alone. Thus, the combination of aspirin plus CDDP may reduce the expression of survivin and induce the apoptosis of SGC7901/CDDP cells.

## Introduction

Chemotherapy is an indispensable component of the comprehensive treatment of gastric cancer. Cisplatin (CDDP) is a widely used chemotherapeutic drug for gastric cancer; however, the resistance of gastric cancer cells to CDDP reduces its therapeutic efficacy. Therefore, there is a need for CDDP-sensitizing agents to enhance the effect of CDDP in the treatment of gastric cancer.

Survivin is a member of the inhibitor of apoptosis protein family (1) that inhibits cell apoptosis and division. The survivin gene is overexpressed in gastric cancer cells (2), which may be one of the main reasons for the resistance to CDDP. It was previously demonstrated that aspirin may enhance the sensitivity of HT-29 human colon cancer cells to 5-FU and a combination of aspirin and 5-FU induces apoptosis of HT-29 cells in a time- and concentration-dependent manner (3). In the present study, we investigated whether aspirin plus CDDP exhibited enhanced toxicity against SGC7901/CDDP cells.

## Materials and methods

### SGC7901 and SGC7901/CDDP cell cultures

SGC7901 cells were obtained from the Cell Bank of Chinese Academy of Sciences (Shanghai, China). The cells were cultured in RPMI-1640 medium (Invitrogen Life Technologies, Carlsbad, CA, USA) supplemented with 10% fetal bovine serum, 100 U/ml penicillin (North China Pharmaceutical Group Corporation, Hebei, China) and 100 μg/ml streptomycin (North China Pharmaceutical Group Corporation) in 75-cm^2^ flasks at 37°C in a humidified atmosphere of 5% CO_2_ and 95% air. The pH value of the medium was adjusted to 7.2 with sterile 5.6% NaHCO_3_ liquid and the medium was changed every 2–3 days. The cells were subcultured when 80% confluence was reached.

As the SGC7901 cells grew to 80% confluence, RPMI-1640 medium with 100 ng/ml CDDP (Qilu Pharmaceutical Co., Ltd., Jinan, China) was added and the medium was changed every 2–3 days. When 80% confluence was reached, the cells were subcultured with RPMI-1640 medium to maintain good cell adhesion. As the cells became adherent to the bottom of the cell culture flasks, RPMI-1640 medium with 200 ng/ml CDDP was added and the culture medium was changed every 2–3 days. The method was repeated with CDDP concentrations of 500, 700 and 1,000 ng/ml, until SGC7901/CDDP cells were obtained. The growth and reproduction of SGC7901/CDDP cells were maintained with RPMI-1640 medium with 1,000 ng/ml CDDP.

The SGC7901/CDDP cells were divided into 4 groups and treated with i) CDDP (10 μg/ml), ii) aspirin (3 mmol/l; Sigma, St. Louis, MO, USA), iii) aspirin (3 mmol/l) plus CDDP (10 μg/ml) and iv) physiological saline. Following incubation for 24 h, the cells were collected with 0.25% trypsin and used in assays measuring viability, apoptosis, survivin mRNA and survivin protein expression.

### MTT cell proliferation assay

The SGC7901/CDDP cells were cultured at a density of 5×10^4^/ml in 96-well plates with 200 μl/well. After adhering overnight at 37°C in a humidified 5% CO_2_ and 95% air atmosphere, the culture solution was aspirated and CDDP (10 μg/ml), aspirin (3 mmol/l), aspirin (3 mmol/l) plus CDDP (10 μg/ml) and physiological saline were added to the respective groups. After 24 h, 20 μl 0.5% MTT solution was added to each well and incubated for 4 h; the culture solution was discarded and 200 μl dimethylsulphoxide was added to each well to dissolve the MTT formazan crystals for 5 min. The absorbance at 490 nm was determined by a multi-detection microplate reader (Sunrise™, Tecan Ltd., Austria). Cell viability was calculated using the following formula: viability=absorbance of the test group-blank/absorbance of the normal group-blank × 100%.

### Flow cytometry

The SGC7901/CDDP cells were centrifuged for 10 min at 1,500 × g and the supernatant was discarded. The cells were washed twice with 4°C phosphate-buffered saline (PBS) solution and resuspended in PBS solution at a concentration of 1.0×10^6^/l. A total of 100 μl solution was collected in 5-ml culture tubes and 5 μl propidium iodide (BD Pharmingen, San Diego, CA, USA) were added. The cells were gently vortexed and incubated for 15 min at room temperature in the dark. A total of 400 μl of 1X binding buffer was added to each tube. Analysis was performed with an EPICS-XL II flow cytometer (Beckman Coulter, Inc., Miami, FL, USA).

### RNA extraction and semiquantitative RT-PCR

Total RNA was extracted using TRIzol reagent (Invitrogen Life Technologies) and cDNA was reverse-transcribed according to the manufacture’s instructions. The 447-bp survivin DNA fragment was amplified using two primers synthesized by Invitrogen Life Technologies, 5′-GCATGGGTGCCCCGACGTTG-3′ and 5′-GCTCCGGCCAGAGGCCTCAA-3′. The PCR reaction was performed in a total volume of 20 μl containing 2 μl 10X PCR buffer, 0.8 μl MgCl_2_, 1.0 μl dNTPs, 0.2 μl of each primer, 2.0 μl cDNA and 1.0 μl Taq DNA polymerase. The amplification conditions were as follows: denaturation at 94°C for 30 sec, annealing at 55°C for 60 sec and elongation at 72°C for 60 sec for 30 cycles. The 241-bp β-actin fragment was amplified using two primers synthesized by Invitrogen Life Technologies, 5′-TAAAGACCTCTATGCCAACACAGT-3′ and 5′-CACCATGGAGGGGCCGGACTCTTC-3′. The PCR reaction was performed in a total volume of 20 μl containing 2 μl 10X PCR buffer, 1.6 μl MgCl_2_, 1.0 μl dNTPs, 0.2 μl of each primer, 2.0 μl cDNA and 1.0 μl Taq DNA polymerase. The amplification conditions were as follows: denaturation at 94°C for 30 sec, annealing at 58°C for 40 sec and elongation at 72°C for 40 sec for 28 cycles. The PCR products were separated on 1% agarose gels containing ethidium bromide. The gel images were digitally recorded and analyzed by computer-assisted image analyzer, Lab-work 4.5 analysis software (Ultra Violet Products, Upland, CA, USA).

### Western blotting

The SGC7901/CDDP cells were washed twice with 4°C PBS. Following the addition of RIPA buffer (Invitrogen Life Technologies), the cells were lysed on ice for 30 min and then clarified by centrifugation at 10,000 × g for 10 min at 4°C. The supernatants were used to assay protein concentration. A total of 25 μg protein were loaded, separated by polyacrylamide gel electrophoresis and transferred onto PVDF membranes. The PVDF membranes were incubated with 5% fat-free powder milk in 500 mmol/l NaCl, 20 mmol/l Tris-HCL (pH 7.5) and 0.5% PBS-Tween-20 for 2 h at room temperature, followed by incubation for 24 h with the appropriate dilutions of primary antibody at 4°C in a refrigerator: 1:2,000 anti-human survivin antibodies (R&D Systems, Minneapolis, MN, USA) and 1:500 β-actin (Wuhan Boster Biological Technology, Ltd., Wuhan, China). Following washing with Tris-buffered saline and Tween-20, the PVDF membranes were incubated with 1:3,000 peroxidase-conjugated rabbit anti-goat sencondary antibodies (Wuhan Boster Biological Technology) for 2 h at room temperature. The proteins were visualized using chemiluminescent peroxidase substrate (Pierce Biotechnology, Inc., Rockford, IL, USA) and the blots were quantified and analyzed by computer-assisted image analyzer, Lab-work 4.5 analysis software.

### Statistical analysis

Data are expressed as means ± SD of at least three independent experiments. One-way ANOVA was used to compare three or more groups. All the analyses were performed with SPSS software, version 19.0 (IBM SPSS, Inc., Chicago, IL, USA). P<0.05 was considered to indicate a statistically significant difference.

## Results

### Cell viability

**Figure 1:**
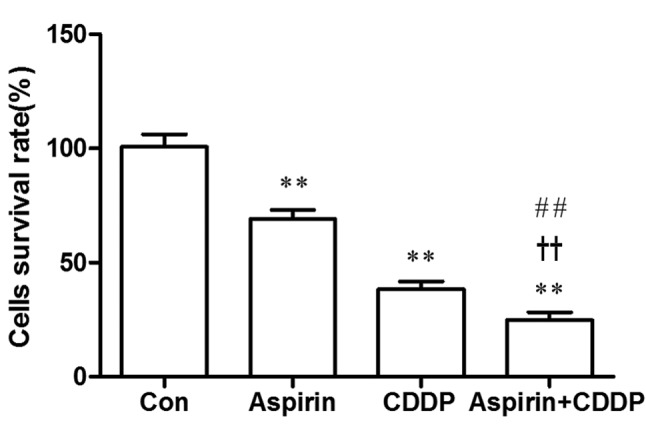
Viability assay of SGC7901/CDDP cells treated with aspirin (3 mmol/l), CDDP (10 μg/ml) and aspirin (3 mmol/l) plus CDDP (10 μg/ml) for 24 h by MTT. Data are presented as mean ± SD, n=6. ^**^P<0.01 vs. untreated SGC7901/CDDP cells (Con). ^##^P<0.01 vs. aspirin (3 mmol/l). ^††^P<0.01 vs. CDDP (10 μg/ml). CDDP, cisplatin.

Compared to the control group, the survival rate of the SGC7901/CDDP cells in the CDDP, aspirin and aspirin plus CDDP groups were lower and the difference was statistically significant (P<0.01). Cell growth was significantly inhibited in the aspirin plus CDDP group and the survival rate of cells in the aspirin plus CDDP was lower compared to that in the CDDP and in the aspirin groups. The difference was statistically significant (P<0.01) (Fig. 1).

### Cell apoptosis

**Figure 2:**
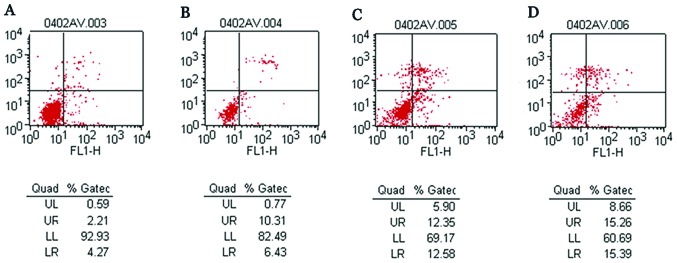
The apoptotic rates were determined by flow cytometry in SGC7901/CDDP cells treated with aspirin (3 mmol/l), CDDP (10 μg/ml) and aspirin (3 mmol/l) plus CDDP (10 μg/ml) for 24 h. (A) Untreated SGC7901/CDDP cells. (B) Aspirin (3 mmol/l). (C) CDDP (10 μg/ml). (D) Aspirin (3 mmol/l) plus cisplatin (10 μg/ml). CDDP, cisplatin.

The apoptotic rate in the aspirin, CDDP, aspirin plus CDDP and control groups was 16.74, 24.93, 30.65 and 6.48%, respectively. Furthermore, the apoptotic rate in the aspirin plus CDDP group was significantly higher compared to that in the other groups (Fig. 2).

### Survivin mRNA expression

**Figure 3:**
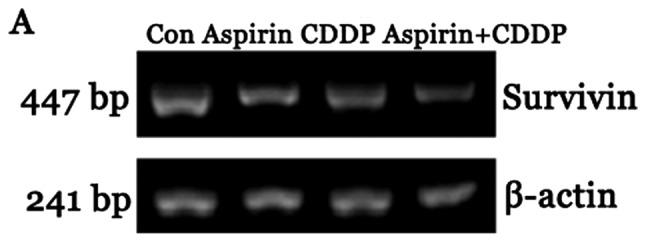

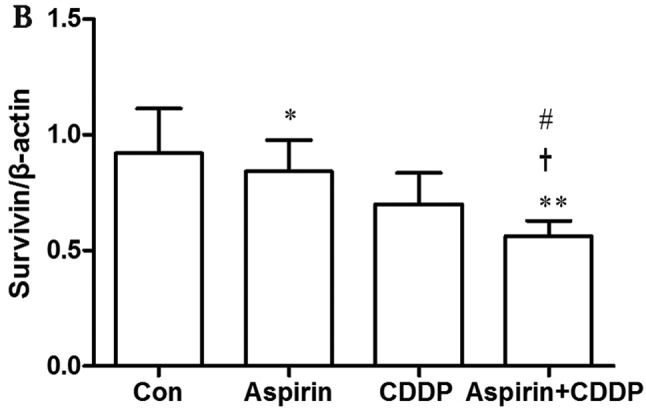
Survivin mRNA expression in SGC7901/CDDP cells treated with aspirin (3 mmol/l), CDDP (10 μg/ml) and aspirin (3 mmol/l) plus CDDP (10 μg/ml) for 24 h by RT-PCR. (A) Representative RT-PCR analysis of survivin expression. (B) Densitometric analysis of survivin mRNA expression. Data are presented as means ± SD, n=5. ^*^P<0.05 vs. untreated SGC7901/CDDP cells (Con). ^**^P<0.01 vs. untreated SGC7901/CDDP cells (Con). ^#^P<0.05 vs. aspirin (3 mmol/l). ^†^P<0.05 vs. CDDP (10 μg/ml). CDDP, cisplatin; Con, control group.

The expression of survivin mRNA in the CDDP and aspirin plus CDDP groups was significantly reduced compared to that in the control group. The difference was statistically significant (P<0.05). Furthermore, the expression of survivin mRNA in the aspirin plus CDDP was significantly reduced compared to that in the aspirin and CDDP alone groups. The difference was statistically significant (P<0.05) (Fig. 3).

### Survivin protein expression

**Figure 4:**
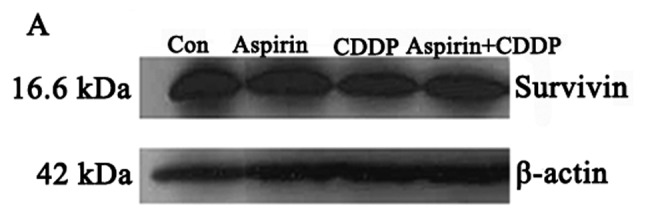

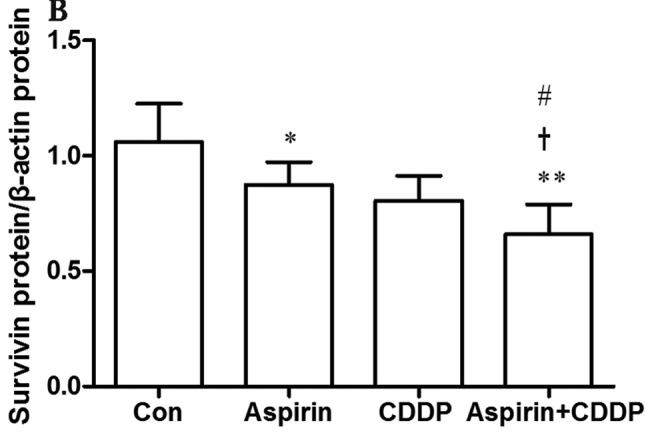
Survivin protein expression in SGC7901/CDDP cells treated with aspirin (3 mmol/l), CDDP (10 μg/ml) and aspirin (3 mmol/l) plus CDDP (10 μg/ml) for 24 h by western blotting. (A) Survivin protein samples obtained from SGC7901/CDDP cells by western blotting. (B) Densitometric analysis of survivin protein expression. Data are presented as means ± SD, n=5. ^*^P<0.05 vs. untreated SGC7901/CDDP cells (Con). ^**^P<0.01 vs. untreated SGC7901/CDDP cells (Con). ^#^P<0.05 vs. aspirin (3 mmol/l). ^†^P<0.05 vs. CDDP (10 μg/ml). CDDP, cisplatin; Con, control group.

The expression of survivin protein in the CDDP and aspirin plus CDDP groups was significantly reduced compared to that in the control group. The difference was statistically significant (P<0.05). Furthermore, the expression of survivin protein in the aspirin plus CDDP group was significantly reduced compared to that in the aspirin and CDDP alone groups. The difference was statistically significant (P<0.05) (Fig. 4).

## Discussion

Gastric cancer is associated with high morbidity and mortality. Approximately 60% of gastric cancer patients in western countries present with advanced-stage disease (4), which is also the case for gastric cancer patients in China (5). Surgery and chemotherapy are currently the mainstay of treatment for patients with advanced gastric cancer. CDDP is one of the most widely used chemotherapeutic agents for the treatment of gastric cancer. However, resistance to CDDP is a major cause of ineffective treatment; therefore, there is a need for CDDP-sensitizing agents to improve the effects of chemotherapy.

Non-steroidal anti-inflammatory drugs (NSAIDs) are widely used in clinical practice due to their antipyretic, analgesic, anti-inflammatory and antirheumatic properties. The antitumor effect of NSAIDs has been extensively investigated (6–8). Li *et al* (9) demonstrated that regular NSAID administration may reduce the incidence of colon cancer by 50% and also reduce the incidence of esophageal and gastric cancer. The apoptosis of cancer cells induced by aspirin may be the mechanism through which aspirin interferes with esophageal carcinogenesis and may be indicative of the potential of NSAIDs as chemopreventive agents in esophageal cancer.

NCX-4016 (a derivative of aspirin containing a nitro group that releases nitric oxide in a sustained fashion for several hours in cells and *in vivo*) combined with CDDP was shown to sensitize drug-resistant strains of human ovarian cancer cells to CDDP. Furthermore, the inhibitory effect of CDDP plus NCX-4016 on drug-resistant strains of human ovarian cancer cells was significantly higher compared to that of CDDP and NCX-4016 alone, indicating that NCX-4016 may enhance the sensitivity of drug-resistant strains of human ovarian cancer cells to CDDP and may specifically eliminate CDDP-refractory cancer cells in patients with recurrent ovarian cancer (10). Kumar and Singh (11) suggested that pre-exposure of tumor cells to aspirin may lower the concentration of CDDP required to exert its cytotoxic effects. This finding may help design novel antitumor protocols with reduced doses of CDDP. Those studies indicate a novel method for overcoming CDDP resistance in the treatment of patients with gastric cancer.

Nakamura *et al* (12) reported a negative correlation between survivin expression in gastric cancer cells and the survival time of patients with gastric cancer receiving CDDP chemotherapy. Those results indicated that survivin may be pivotal in the development of gastric cancer and resistance to CDDP and, therefore, controlling the expression of the survivin gene may be useful in the chemotherapy of gastric cancer, a hypothesis also supported by other studies (13,14). The abovementioned results indicated that the survivin gene is closely associated with resistance to CDDP in the chemotherapy of gastric cancer.

In our experiments, the results of the MTT assay demonstrated that the cell survival rate in the aspirin plus CDDP group was significantly reduced. The flow cytometry test results revealed that the apoptotic rate in the aspirin plus CDDP group was significantly higher compared to that in the other groups. In addition, the RT-PCR and western blotting results revealed that the expression of survivin mRNA and protein were significantly reduced in the aspirin plus CDDP group. Taken together, these experimental results indicate that aspirin plus CDDP may exhibit significantly enhanced toxicity against SGC7901/CDDP cells compared to aspirin or CDDP alone, possibly through reducing survivin expression and inducing the apoptosis of SGC7901/CDDP cells.

Related research demonstrated that NSAIDs may induce the apoptosis of tumor cells through a COX-2 non-dependent pathway and exert antitumor effects (15,16). Shao *et al* (17) suggested that the inhibition of NFκB activity is a plausible mechanism for apoptosis induced by the wild-type p53 gene transfer in human colon cancer cells and that anti-NFκB reagent aspirin may render these cells more susceptible to apoptosis. Adachi *et al* (18) suggested that increased ROS generation is one of the key mechanisms underlying the NSAID-mediated anticancer effects on various types of cancer cells. Oh *et al* (19) reported that the enhancement of mitochondrial permeability transition-dependent apoptosis by salicylates may be the mechanism underlying the protective effect of aspirin and other NSAIDs against colon, lung and breast cancers. Pathi *et al* (20) also suggested that the anticancer activity of aspirin may be due to its salicylate metabolite.

Our results were obtained by aspirin (3 mmol/l) plus CDDP (10 μg/ml) acting on SGC7901/CDDP cells for 24 h. However, further investigation is required to determine whether the increased toxicity is dose- and time-dependent and whether there are additional mechanisms underlying the increased toxicity exhibited by aspirin plus CDDP against SGC7901/CDDP cells.
